# Mid‐childhood outcomes of infant siblings at familial high‐risk of autism spectrum disorder

**DOI:** 10.1002/aur.1733

**Published:** 2016-11-29

**Authors:** Elizabeth Shephard, Bosiljka Milosavljevic, Greg Pasco, Emily J. H. Jones, Teodora Gliga, Francesca Happé, Mark H. Johnson, Tony Charman, The BASIS Team

**Affiliations:** ^1^ MRC Social, Genetic & Developmental Psychiatry CentreInstitute of Psychiatry, Psychology, & Neuroscience, King's College LondonLondonUK; ^2^ Department of PsychologyInstitute of Psychiatry, Psychology, & Neuroscience, King's College LondonLondonUK; ^3^ Centre for Brain and Cognitive Development, School of PsychologyBirkbeck CollegeLondonUK

**Keywords:** high‐risk siblings, clinical outcomes, ADHD, anxiety, broader autism phenotype

## Abstract

Almost 20% of infants with an older sibling with autism spectrum disorder (ASD) exhibit ASD themselves by age 3 years. The longer‐term outcomes of high‐risk infants are less clear. We examined symptoms of ASD, attention‐deficit/hyperactivity disorder (ADHD) and anxiety, language, IQ, and adaptive behaviour at age 7 years in high‐ and low‐risk children prospectively studied since the first year of life. Clinical outcomes were compared between high‐risk children who met diagnostic criteria for ASD at age 7 (HR‐ASD‐7 group, *n* = 15), high‐risk children without ASD (HR‐Non‐ASD‐7 group, *n* = 24), and low‐risk control children (LR group, *n* = 37). Diagnostic stability between age 3 and 7 years was moderate, with five children who did not meet diagnostic criteria for ASD at age 3 years being assigned the diagnosis at age 7, and three children showing the opposite pattern. The HR‐ASD‐7 group showed elevated ADHD and anxiety symptoms and had lower adaptive behaviour scores than LR controls. The HR‐Non‐ASD‐7 group had higher repetitive behaviour, lower adaptive functioning and elevated scores on one anxiety subscale (Separation Anxiety) compared to LR controls, but evidence for subclinical ASD symptoms (the broader autism phenotype, BAP) was limited in the group as a whole, although we identified a subgroup with elevated ASD traits. The difficulties experienced by high‐risk siblings at school‐age extend beyond ASD symptoms. The pattern of difficulties exhibited by the HR‐ASD‐7 group may inform our understanding of developmental trajectories of co‐occurring psychopathology in ASD. ***Autism Res*** *2017, 10: 546–557*. © The Authors Autism Research published by Wiley Periodicals, Inc. on behalf of International Society for Autism Research

## Introduction

Prospective studies of infants at familial high‐risk of autism spectrum disorder (ASD) because of having an older sibling with ASD are predicated on the known elevated recurrence rate of ∼10% [Constantino, Zhang, Frazier, Abbacchi, & Law, 2010; Sandin et al., 2014] compared to the general population prevalence of ∼1% [Baird et al., 2006; CDC, 2014]. In prospective high‐risk sibling studies the recurrence rates have been higher, with close to 20% of high‐risk infants meeting diagnostic criteria for ASD when assessed at age 3 years [Messinger et al., 2015; Ozonoff et al., 2011]. In addition, a further ∼20% of non‐ASD high‐risk siblings show elevated ASD severity scores and/or lower levels of developmental ability when assessed at the same age [Charman et al., 2016; Messinger et al., 2013].

Ozonoff et al. [2014] found that around a quarter of high‐risk siblings who did not have ASD were “non‐typically developing” at age 3 based on scoring above threshold on the Autism Diagnostic Observational Schedule [ADOS; Lord et al., 2000] and/or low scores on the Mullen Scales of Early Learning [MSEL; Mullen, 1995]. Clinical judgement of these children found that around one third showed aspects of the “broader autism phenotype” (BAP)—subclinical social‐communication and restricted, repetitive behaviour atypicalities seen at an elevated rate in family members of individuals with ASD [Bolton et al., 1994; Pickles et al., 2000; Piven, Palmer, Jacobi, Childress, & Arndt, 1997]—while others showed features of emergent attention‐deficit/hyperactivity disorder (ADHD), general developmental delay, or speech and language difficulties [Ozonoff et al., 2014]. In an overlapping sample, Miller, Young, et al. [2015] reported elevated rates of pragmatic language difficulties and Schwichtenberg et al. [2013] slightly elevated anxiety, depression, and aggression scores in high‐risk non‐ASD siblings at 3 years. The emerging pattern from these studies suggests that, from a young age, high‐risk children are vulnerable to developing not only ASD and subclinical ASD traits, but also problems with language [Pickles, Anderson, & Lord, 2014; Wodka, Mathy, & Kalb, 2013], ADHD and anxiety [Simonoff et al., 2008; White, Oswald, Ollendick, & Scahill, 2009] that frequently co‐occur with ASD and overlap at the genetic level [Hallett et al., 2013; Ronald, Simonoff, Kuntsi, Asherson, & Plomin, 2008; Tick et al., 2016].

Recently, groups have reported on clinical outcomes in high‐risk siblings followed further, into the school‐age years. Gamliel et al. [2009] reported that 41% of high‐risk siblings without ASD showed either low scores on IQ, language or attainment measures and/or parent reported problems in social‐communication and stereotyped behaviours at age 7 years. Subsequently, Ben‐Yizhak et al. [2011] reported this subgroup of high‐risk siblings to display elevated “semantic‐pragmatic” language difficulties compared to typically developing high‐ and low‐risk siblings. In contrast, Gillespie‐Lynch et al. [2015] reported difficulties with structural and pragmatic aspects of language development at age 7 only in high‐risk siblings who themselves had ASD, and not in those without ASD, compared to low‐risk controls. Similarly, Drumm, Bryson, Zwaigenbaum, and Brian [2015] found that non‐ASD high‐risk siblings showed average/above‐average expressive, receptive, and pragmatic language abilities at 8–11 years, although pragmatic language was relatively weaker than structural language, and the siblings showed impairments in phonological aspects of language. Warren et al. [2012] reported on outcomes at age 5 years in non‐ASD high‐risk siblings and found few differences compared to low‐risk controls on global measures of IQ, language, and emotional and behavioural problems, but slightly elevated social cognition difficulties and restricted and repetitive behaviours. Miller, Iosif, et al. [2015] reported higher internalising and externalising problems and poorer receptive and expressive language abilities in a group of high‐risk non‐ASD siblings with clinical concerns (BAP, ADHD‐like problems, emotional problems, developmental delay) compared to typically developing high‐ and low‐risk siblings, who did not differ from one another, at 5–9 years.

In summary, school‐age follow‐up studies indicate that ASD traits and language, behavioural and emotional problems persist into mid‐childhood for some high‐risk siblings who do not develop ASD. However, there is some inconsistency in which domains are affected, perhaps reflecting heterogeneity in the development of high‐risk non‐ASD infants, or the restricted range of clinical outcomes (e.g., only language) examined in many studies. Another issue is that high‐risk infants who do develop ASD have largely been excluded from analyses in school‐age follow‐up studies [e.g., Drumm et al., 2015; Miller, Iosif, et al., 2015; Warren et al., 2012]. This precludes the comparison of levels of co‐occurring difficulties such as ADHD, anxiety and language problems between high‐risk siblings who develop ASD and those who do not.

To date, only one study has reported on diagnostic stability of ASD into the school‐age years in high‐risk children. Brian et al. [2015] reported that 17/18 (94.4%) high‐risk toddlers with ASD continued to meet diagnostic criteria for ASD at 7–12 years while one child no longer met ASD criteria, and 6/49 (12.2%) non‐ASD high‐risk toddlers were classified as “Later‐diagnosed” due to meeting diagnostic criteria for ASD in mid‐childhood but not at age 3 years (*pace* DSM‐5, which allows symptoms to date from “the developmental period”). However, Brian et al. [2015] did not report on other developmental outcomes, and so the question of what other difficulties, such as co‐occurring ADHD and anxiety, high‐risk children with ASD experience remains to be answered.

The current study reports on a wide range of mid‐childhood clinical outcomes as well as the stability of ASD diagnosis between age 3 and 7 years in a cohort of high‐risk children, including both those who met diagnostic criteria for ASD at age 7 years (HR‐ASD‐7) and those who did not (HR‐Non‐ASD‐7). Intelligence, language ability, adaptive behaviour, and symptoms of ADHD and anxiety were compared between the two high‐risk subgroups and a group of low‐risk (LR) children to assess differences in mid‐childhood clinical outcomes. Given sex differences in ASD symptomatology and developmental ability recently reported in high‐risk siblings in early childhood, with boys more severely affected than girls [Messinger et al., 2015], and known sex differences in rates of ADHD (males > females) and anxiety (females > males) in non high‐risk samples [Arnett, Pennington, Willcutt, DeFries, & Olson, 2015; Lewinsohn, Gotlib, Lewinsohn, Seeley, & Allen, 1998], we explored whether sex influenced school‐age outcomes. We predicted the following pattern of findings:

1. The HR‐ASD‐7 group would show lower IQ, language ability and adaptive functioning, and higher symptoms of ADHD and anxiety compared to the HR‐Non‐ASD‐7 group and LR controls.
2. In line with the BAP concept, the HR‐Non‐ASD‐7 group would show elevated ASD traits compared to the LR controls. These children may also show lower levels of IQ, language and adaptive behaviour and higher levels of ADHD and anxious traits than the LR controls.
3. Boys would show higher ASD and ADHD symptomatology than girls, while girls would show higher levels of anxiety than boys.

## Methods

### Participants

Participants were 104 children taking part in a prospective longitudinal study of infants at high‐ and low‐familial risk for autism (hereafter, HR and LR) recruited as part of the British Autism Study of Infant Siblings (BASIS; www.basisnetwork.org). Siblings completed research visits at 7 and 14 months of age, around their second and third birthdays, and were invited to return for the current follow‐up study at age 6–8 years. At enrolment, each HR infant (*n* = 54) had an older sibling (in 4 cases, a half‐sibling) with a community clinical ASD diagnosis, confirmed using information from the *Development and Well‐Being Assessment* [*DAWBA*; Goodman, Ford, Richards, Gatward, & Meltzer, 2000] and the *Social Communication Questionnaire* [*SCQ*; Rutter, Bailey, & Lord, 2003] by expert clinicians (TC, PB).^1^ Parent‐reported family medical histories were examined for significant conditions in the proband or extended family members (e.g., Fragile X syndrome, tuberous sclerosis) with no such conditions reported. LR controls (*n* = 50) were full‐term infants (with one exception) recruited from a volunteer database at the Birkbeck Centre for Brain and Cognitive Development. Medical history review confirmed lack of ASD within first‐degree relatives. At enrolment, all LR infants had at least one older sibling. The SCQ was used to confirm absence of ASD in these older siblings, with no child scoring above instrument cut‐off (≥ 15; *n* = 1 missing data).

Of 53 HR and 48 LR children retained at the 3 year assessment, 44 HR (83%) and 37 LR (77%) agreed to take part in the follow‐up study at 6–8 years. Of these, two HR children did not complete a research visit (parents completed questionnaires only) and we were unable to assign them to an ASD outcome group and consequently excluded them from analysis, leaving a final sample of 42 HR siblings (15 boys, 27 girls) and 37 LR controls (15 boys, 22 girls). The HR and LR groups did not differ in age (HR mean (SD): 90.6 (6.3) months; LR mean (SD): 89.3 (4.9) months; *t* (75) = −.96, *P* = .34) or sex (HR % male: 35.7; LR % male: 40.5; *χ^2^* (1) = .20, *P* = .66) at the follow‐up. The retained sample did not differ from the non‐retained sample in 3 year levels of ASD on the ADOS, SRS, or SCQ, developmental level on the MSEL, adaptive behaviour assessed with the *Vineland Adaptive Behavior Scales—Second Edition* [*Vineland‐II*; Sparrow, Balla, & Cicchetti, 2005], or family income (all *P* > .4). Ethical approval was obtained from the NHS National Research Ethics Service (NHS RES London REC 14/LO/0170). Parents provided written informed consent. Children provided written informed assent wherever possible given developmental level.

### Outcome Measures Collected at 7 Years

*ASD symptomatology*. The *Autism Diagnostic Observation Schedule—Second Edition* [*ADOS‐2*; Lord et al., 2012], a standardised interaction observation assessment, was used to assess current symptoms of ASD (module 3 for 73 children, module 2 for one child, module 1 for one child; ADOS not completed with 3 LR children). Calibrated Severity Scores for Social Affect, Restricted and Repetitive Behaviours (RRB), and Overall Total were computed [Gotham, Pickles, & Lord, 2009; Hus, Gotham, & Lord, 2014], which provide standardised autism severity measures that account for differences in module administered, age and verbal ability. The *Autism Diagnostic Interview—Revised* [*ADI‐R*; Le Couteur, Lord, & Rutter, 2003], a structured parent interview, was completed with parents of HR children. Standard algorithm scores were computed for Reciprocal Social Interaction (Social), Communication, and Restricted, Repetitive and Stereotyped Behaviours and Interests (RRB).

Parents completed questionnaires to obtain further information on ASD symptoms and subclinical ASD traits. The *Social Responsiveness Scale—Second Edition* [*SRS‐2*; Constantino, 2012] assessed parent‐rated social impairments over the past 6 months and age‐and sex‐normed T‐scores (mean 50; SD 10) were used in analysis. The *Short Sensory Profile* [*SSP*; Dunn, 1999] assessed parent‐rated sensory processing difficulties; the total SSP score was used in analysis. The *Repetitive Behavior Scale—Revised* [*RBS‐R*; Bodfish, Symons, & Lewis, 1999] measured parent‐rated repetitive/restricted behaviours; the total RBS‐R score was used in analysis. Higher scores on the SRS‐2 and RBS‐R reflect more severe ASD symptoms; lower scores on the SSP reflect greater sensory processing problems.

### IQ and Adaptive Behaviour

The *Wechsler Abbreviated Scale of Intelligence—Second Edition* [*WASI‐II*; Wechsler, 2011], a standardised instrument to assess intellectual ability, was completed with each child. Age‐normed intelligence quotients (mean 100; SD 15) for the verbal domain (Verbal Comprehension Index, VCI), performance domain (Perceptual Reasoning Index, PRI), and full‐scale IQ (FSIQ) were used in analyses. One HR child was unable to complete the assessment due to intellectual disability.

Adaptive behaviour was assessed using the Vineland‐II [Sparrow et al., 2005], a semi‐structured interview in which parents rate their child's current level of functioning across the domains of Communication, Daily Living, and Socialization. Age‐normed Standard Scores (mean 100; SD 15) were computed for each domain and the Adaptive Behaviour Composite (ABC).

### Language

The *Clinical Evaluation of Language Fundamentals—Fourth Edition UK* [*CELF‐4*; Semel, Wiig, & Secord, 2006] is an age‐normed standardised instrument to assess language ability. The Concepts and Following Directions and Recalling Sentences subtests were completed with HR and LR children to assess receptive and expressive language, respectively (mean 10; SD 3). Two HR children had insufficient language to complete the assessment; a further four HR and 10 LR children did not complete the assessment due to time constraints. Standard Scores on the Communication domain of the Vineland‐II were used as an additional measure of language ability.

### ADHD and Anxiety

The parent‐rated *Conners 3* [Conners, 2008] was used to assess symptoms of ADHD over the past 6 months. T‐scores (mean 50; SD 10) for the DSM‐IV‐TR Inattentive and Hyperactive/Impulsive scales were used in analyses. The parent‐report *Spence Children's Anxiety Scale* [*SCAS*; Spence, 1998] was used to assess current symptoms of children's anxiety. The SCAS is based on DSM‐IV‐TR [American Psychiatric Association, 2000] criteria for anxiety disorders and generates scores for the domains of Separation Anxiety, Social Phobia, Obsessive Compulsive Disorder (OCD), Physical Injury Fears, Panic/Agoraphobia, and Generalized Anxiety, as well as a Total Anxiety score. Scores on the six anxiety domains and the total SCAS score were used in analyses. We were also interested in how many children in each group showed ADHD and anxiety scores in the clinically significant range. We therefore calculated the percentage of children per group with T‐scores > 65 on the Conners [T‐scores over 65 are considered to be indicative of clinically significant symptoms, Conners, 2008] and the percentage of children with z‐transformed SCAS Total Anxiety scores 1.5 SD above the mean of a large (*n* = 261) normative sample of typically developing 6‐11 year olds [Nauta et al., 2004].

### Assignment to ASD Outcome Groups at 3 and 7 Years

*3 years*: Experienced researchers who conducted the assessments (KH, SC, GP) and the lead clinician (TC) reviewed information across the research visits at 2 years (including ADOS‐G, MSEL, and Vineland assessments) and 3 years (including MSEL, Vineland, ADOS‐G, and ADI‐R) and as a team assigned clinical consensus best estimate diagnosis of ASD according to ICD‐10 [World Health Organisation, 1993]. For LR control children, in the absence of a full developmental history (no ADI‐R was administered) no formal clinical diagnoses were considered but none had a community clinical ASD diagnosis.

*7 years*: Experienced researchers who conducted the assessments (ES, BM, GP) and the lead clinician (TC) reviewed information on ASD symptomatology (ADOS‐2, ADI‐R (HR only), SCQ), adaptive functioning (Vineland‐II), and IQ (WASI‐II) for each HR and LR child and as a team assigned clinical consensus best estimate diagnosis of ASD according to DSM‐5 [American Psychiatric Association, 2013]. None of the 37 LR children met DSM‐5 criteria for ASD and none had a community clinical ASD diagnosis.

Diagnosis at age 7 years included review of all information previously obtained, including from the 2 year and 3 year visits. Note that there was overlap in the personnel involved in the diagnostic decision‐making (GP, TC) so the decisions were not independent. However, the age 7 diagnostic decisions were not directly “yoked” to the diagnostic decisions previously taken at the 3 year visit but rather, in light of the large amount of additional information available about the children—in particular as by school age the ADI‐R includes more information (e.g., with respect to peer interactions; functioning outside the home setting) and uses the full set of diagnostic algorithm items which are not all applicable at 3 years—a decision was made as to whether the child currently met DSM‐5 criteria for ASD. In line with the primary focus of the current paper on mid‐childhood outcomes in HR siblings, and not diagnostic stability over time per se, we report findings in relation to 7 year ASD diagnosis made on the basis of information available from all clinical assessments up to that timepoint.

### Statistical Analysis

All statistical analyses were conducted in SPSS v21 [IBM Corp, [Link]]. The hypothesised group differences in 7 year outcomes were assessed using ANOVA or MANOVA tests with group (HR‐ASD‐7, HR‐Non‐ASD‐7, LR) as a between‐subjects factor. Measures with one dependent variable (SRS‐2, RBS‐R, SSP) were analysed using univariate ANOVAs. Measures with multiple dependent variables (ADOS‐2, ADI‐R, WASI‐II, Vineland II, CELF, Conners 3, SCAS) were analysed using MANOVAs. Significant group effects were further investigated with planned contrasts between each pair of groups with Tukey's HSD correction applied to control for multiple comparisons. Sex differences in school‐age outcomes and sex‐by‐group interactions were investigated using 2 (Group: LR, HR) x 2 (Sex: male, female) ANOVA/MANOVAs.

Following previous school‐age follow‐up studies [Gamliel et al., 2009; Miller, Iosif, et al., 2015], we conducted supplementary analyses to explore differences in key outcome variables (ASD, IQ, adaptive behaviour, language, ADHD, anxiety) between HR non‐ASD children who showed signs of atypical development, defined as scoring above clinical cutoffs for ASD on either the ADOS‐2 or the ADI‐R, and those with typical development (see Supporting Information).

## Results

### Diagnostic Stability from 3 to 7 Years

At age 7 years, 15 HR children (7 boys, 8 girls) met DSM‐5 [APA, 2013] criteria for ASD and the remaining 27 children (8 boys, 19 girls) did not. Of the 13 HR siblings with an ASD diagnosis at 3 years also seen at 7 years, 10 retained their ASD diagnosis (76.9%) and 3 (23.1%) did not (“lost diagnosis”—see Discussion). Of the 29 HR who were not given an ASD diagnosis at 3 years also seen at 7 years, 24 did not meet diagnostic criteria for ASD (82.8%) at 7 years but 5 (17.2%) did meet ASD criteria (“later diagnosed”). Scores for the HR groups broken down by both 3‐year and 7‐year diagnostic outcome are shown in the Supporting Information.

### Group Characteristics and ASD Symptomatology at 7 Years

The 15 HR children meeting DSM‐5 criteria for ASD at age 7, including the 5 “later diagnosed” children, formed the HR‐ASD‐7 group. Of the 27 HR children who did not meet ASD criteria at age 7, the 3 “lost diagnosis” children were excluded from further analysis (given they met ICD‐10 criteria for ASD earlier in development), leaving 24 HR children in the HR‐Non‐ASD‐7 group. Group characteristics, ASD symptomatology, and statistical test results are displayed in Table 1; Cohen's *d* effect sizes for pairwise group contrasts are given in Table S3 (Supporting Information). The groups did not differ in age or sex at the 7 year follow‐up. As expected, the groups differed significantly on ADI‐R, ADOS‐2, SRS‐2, RBS‐R, and SSP measures of ASD symptoms. The HR‐ASD‐7 group had higher ADI‐R scores than the HR‐Non‐ASD‐7 group (all *P* < .001, all *d* ≥ 1.33), higher ADOS‐2 Social Affect, RRB, and Total CSS than the LR controls (all *P* ≤ .001, all *d* ≥ 2.02), and higher SRS‐2 and RBS‐R and lower (more atypical) SSP scores than the HR‐Non‐ASD‐7 group and LR controls (*P* = .004, *d* = .85). The HR‐Non‐ASD‐7 group had higher ADOS‐2 RRB CSS than the LR group (*P* = .003, *d* = .93).

**Table 1:** Group Characteristics and ASD Symptomatology at 7 Years

7‐Year Outcome Measure	LR *N* = 37*	HR‐Non‐ASD‐7 *N* = 24*	HR‐ASD‐7 *N* = 15*	MANOVA/ANOVA Group effects
*Age (months)*	89.34 (4.81)	91.42 (6.28)	89.13 (6.53)	n/s
*Sex (male: female)*	15:22	5:19	7:8	n/s
*ADI*—*Social*	Not completed	4.04 (5.48)^a^	13.14 (4.69)^b^	*F*(1, 36) = 27.00, *P* < .001, *η^*2*^* = .429
*ADI*—*Communication*	Not completed	4.25 (4.67)^a^	10.43 (4.59)^b^	*F*(1, 36) = 15.70, *P* < .001, *η^*2*^* = .304
*ADI*—*RRB*	Not completed	0.58 (1.41)^a^	3.57 (1.74)^b^	*F*(1, 36) = 33.33, *P* < .001, *η^*2*^* = .481
*ADOS*—*CSS Total*	1.70 (1.19)^a^	2.46 (1.41)	6.33 (2.92)^b^	*F*(2, 69) = 37.61, *P* < .001, *η^*2*^* = .522
*ADOS CSS SA*	2.18 (1.70)^a^	2.96 (1.60)	6.60 (2.59)^b^	*F*(2, 69) = 29.11, *P* < .001, *η^*2*^* = .458
*ADOS CSS RRB*	1.12 (0.70)^a^	3.04 (2.84)^b^	6.13 (2.70)^b^	*F*(2, 69) = 29.80, *P* < .001, *η^*2*^* = .463
*SRS‐2*	45.49 (5.82)^a^	52.37 (11.74)^a^	74.85 (22.77)^b^	*F*(2, 64) = 26.59, *P* < .001, *η^*2*^* = .454
*RBS‐R*	1.40 (2.05)^a^	5.05 (11.54)^a^	30.08 (33.55)^b^	*F*(2, 65) = 16.15, *P* < .001, *η^*2*^* = .332
*SSP*	174.09 (11.49)^a^	167.38 (22.83)^a^	141.58 (36.26)^b^	*F*(2, 64) = 10.31, *P* < .001, *η^*2*^* = .244

Means (SD) are presented by ASD outcome group.

Groups marked with different superscript letters (a, b, c) differed significantly with Tukey's HSD correction applied (*P* < .05).

*Group sizes are smaller for some variables due to missing data.

HR‐ASD‐7 group minimum *n* = 12; HR‐Non‐ASD‐7 group minimum *n* = 19; LR group minimum *n* = 33.

ADI, autism diagnostic interview—revised; RRB, repetitive and restrictive behaviour; ADOS, autism diagnostic observation schedule; CSS, calibrated severity score; SA, social affect; SRS‐2, social responsiveness scale—2; RBS‐R, repetitive behavior scale—revised; SSP, short sensory profile.

### IQ, Adaptive Behaviour, and Language at 7 Years

WASI‐II FSIQ differed significantly between groups, reflecting marginally lower scores in the HR‐Non‐ASD‐7 group than the LR controls (*P* = .05, *d* = .75), although all groups' scores were in the typical range (Table 2). WASI‐II VCI and PRI scores did not differ between groups. The groups differed significantly on all adaptive behaviour variables (Table 2). The HR‐ASD‐7 group had lower ABC, Socialization, and Daily Living scores than the HR‐Non‐ASD‐7 and LR groups (all *P* ≤ .005, all *d* ≥ .85). The HR‐Non‐ASD‐7 group had lower ABC and Daily Living scores than the LR controls (all *P* ≤ .04, all *d* ≥ .77). For language variables, the groups differed significantly on Vineland Communication, with lower scores in the HR‐ASD‐7 group than the LR group (*P* = .02, *d* = .81), but not in receptive or expressive performance on the CELF (Table 2).

**Table 2:** IQ, Language, and Adaptive Behaviour Outcomes at 7 Years

7‐Year Outcome Measure	LR *N* = 37*	HR‐Non‐ASD‐7 *N* = 24*	HR‐ASD‐7 *N* = 15*	MANOVA/ANOVA Group effects
*WASI‐II FSIQ*	117.06 (11.61)^a^	107.96 (12.76)^b^	109.79 (21.36)	*F*(2, 70) = 3.25, *P* = .05, *η^*2*^* = .085
*WASI‐II VCI*	119.77 (13.93)	110.83 (14.94)	110.14 (25.87)	n/s
*WASI‐II PRI*	110.34 (12.05)	102.71 (9.97)	109.57 (18.26)	n/s
*Vineland ABC*	110.53 (6.98)^a^	102.22 (12.67)^b^	90.27 (15.46)^c^	*F*(2, 69) = 17.66, *P* < .001, *η^*2*^* = .339
*Vineland Socialization*	110.29 (5.27)^a^	104.65 (11.62)^a^	84.00 (17.49)^b^	*F*(2, 69) = 30.78, *P* < .001, *η^*2*^* = .471
*Vineland Daily Living*	105.94 (9.28)^a^	98.38 (10.26)^b^	86.07 (15.65)^c^	*F*(2, 69) = 16.48, *P* < .001, *η^*2*^* = .323
*Vineland Comm*.	116.62 (11.09)^a^	111.88 (14.50)	104.53 (18.08)^b^	*F*(2, 69) = 4.21, *P* = .02, *η^*2*^* = .109
*CELF CFD*	12.71 (2.20)	11.85 (3.05)	10.83 (3.38)	n/s
*CELF RS*	12.79 (3.01)	11.30 (2.13)	12.55 (2.77)	n/s

Group means (SD) are presented by ASD outcome group.

Groups marked with different superscript letters (a, b, c) differed significantly with Tukey's HSD correction applied (*P* < .05).

*Group sizes are smaller for some variables due to missing data.

HR‐ASD‐7 group minimum *n* = 11 (CELF variables), *n* = 14 (other variables); HR‐Non‐ASD‐7 group minimum *n* = 20 (CELF), *n* = 23 (other variables); LR group minimum *n* = 24 (CELF), *n* = 34 (other variables).

WASI‐II FSIQ, VCI, PRI, Wechsler Abbreviated Scale of Intelligence—II Full‐Scale IQ, Verbal Comprehension Index, Perceptual Reasoning Index. Vineland ABC, Socialisation, Daily Living, Comm., Vineland Adaptive Behavior Scales—II Adaptive Behavior Composite (ABC) and Standard Scores for Socialization, Daily Living and Communication domains. CELF CFD RS, Clinical Evaluation of Language Fundamentals—4^th^ Edition Scaled Scores for Concepts and Following Directions (CFD) and Recalling Sentences (RS).

### ADHD and Anxiety at 7 Years

The groups differed significantly in Conners Hyperactive/Impulsive and Inattentive T‐scores (Table 3). The HR‐ASD‐7 group had higher scores on both ADHD domains than the LR controls (both *P* ≤ .007, both *d* ≥ .90), while the remaining group pairs did not differ. MANOVA on SCAS anxiety scores revealed significant group differences in Total Anxiety and most subscales (Table 3). The HR‐ASD‐7 group had significantly higher scores than LR controls for Separation Anxiety, OCD, Panic/Agoraphobia, Generalized Anxiety, and Total Anxiety (all *P* ≤ .02, *d* ≥ .64), and higher Panic/Agoraphobia scores than the HR‐Non‐ASD‐7 group (*P* = .01, *d* = .66). The HR‐Non‐ASD‐7 group had higher Separation Anxiety scores than the LR controls (*P* = .04, *d* = .77). The HR‐ASD‐7 group had the largest proportion of children scoring above clinical cut‐offs for the Conners ADHD domains and SCAS Total Anxiety, followed by the HR‐Non‐ASD‐7 group and then the LR group. Due to small numbers of LR children scoring above threshold, these proportional measures were not analysed statistically.

**Table 3:** ADHD and Anxiety Scores at 7 Years

7‐year Outcome Measure	LR *N* = 37*	HR‐Non‐ASD‐7 *N* = 24*	HR‐ASD‐7 *N* = 15*	MANOVA/ANOVA Group effects
*Conners Hyp/Imp*	52.16 (11.58)^a^	57.05 (16.10)	67.73 (15.58)^b^	*F*(2, 71) = 6.73, *P* = .002, *η^*2*^* = .159
*% Hyp/Imp T‐score ≥ 65*	13.51%	27.27%	53.00%	–
*Conners Inattentive*	51.22 (9.40)^a^	56.50 (12.90)	62.40 (14.91)^b^	*F*(2, 71) = 5.08, *P* = .009, *η^*2*^* = .125
*% Inattentive T‐score ≥ 65*	10.81%	22.73%	33.33%	–
*SCAS Separation*	2.94 (2.14)^a^	4.87 (2.82)^b^	6.27 (4.20)^b^	*F*(2, 71) = 8.01, *P* = .001, *η^*2*^* = .184
*SCAS OCD*	0.67 (0.99)^a^	1.00 (1.51)	2.27 (3.41)^b^	*F*(2, 71) = 3.94, *P* = .02, *η^*2*^* = .100
*SCAS Social Phobia*	2.64 (2.98)	4.13 (2.67)	5.07 (5.65)	n/s
*SCAS Physical Injury*	2.97 (2.08)	3.43 (2.25)	4.53 (2.85)	n/s
*SCAS Panic/Agor*.	0.53 (0.94)^a^	0.74 (0.97)^a^	2.93 (4.62)^b^	*F*(2, 71) = 6.60, *P* = .002, *η^*2*^* = .157
*SCAS Generalised*	2.47 (1.40)^a^	3.74 (2.05)	5.13 (3.66)^b^	*F*(2, 71) = 8.00, *P* = .001, *η^*2*^* = .184
*SCAS Total Score*	12.22 (7.27)^a^	17.91 (8.55)	26.20 (20.86)^b^	*F*(2, 71) = 7.84, *P* = .001, *η^*2*^* = .181
*% Total SCAS score 1.5 SD ≥ TD mean*	2.78%	8.70%	26.67%	*–*

Group means (SD) are presented by ASD outcome group.

Groups marked with different superscript letters (a, b, c) differed significantly with Tukey's HSD correction applied (*P* < .05).

*Group sizes are smaller for some variables due to missing data.

HR‐ASD‐7 group minimum *n* = 15; HR‐Non‐ASD‐7 group minimum *n* = 22; LR group minimum *n* = 36. *Conners Hyp/Imp and Inattentive* = Conners 3 T‐scores for Hyperactive/Impulsive and Inattentive symptoms; mean T‐scores per group and the percentage of each group with T‐scores ≥ 65 are presented (percentage measures not analysed due to small *n*s and cell counts < 5).

SCAS, Spence Children's Anxiety Scale (parent‐rated scores for Separation Anxiety, obsessive‐compulsive disorder (OCD), Social Phobia, Physical Injury Fears, Panic/Agoraphobia, Generalised Anxiety, and Total Anxiety). % Total SCAS score 1.5 SD ≥ TD mean = percentage of children per group scoring 1.5 SD above the mean score for children without anxiety disorders after z‐transformation.

### Sex Differences in Outcome Variables

There were significant effects of sex on ADOS‐2 Total (*F*(1, 68) = 7.53, *P* = .008, *η^2^* = .100), Social Affect (*F*(1, 68) = 7.28, *P* = .009, *η^2^* = .097) and RRB (*F*(1, 68) = 4.38, *P* = .04, *η^2^* = .060) CSS, Vineland Communication (*F*(1, 68) = 6.51, *P* = .01, *η^2^* = .087), and Generalised Anxiety (*F*(1, 70) = 4.07, *P* = .05, *η^2^* = .055). Boys had significantly higher ADOS scores and lower Communication and Generalised Anxiety than girls across HR and LR groups. There was a significant group‐by‐sex interaction for FSIQ (*F*(1, 69) = 5.52, *P* = .03, *η^2^* = .074), reflecting a trend for higher scores in girls than boys in the LR group only.

## Discussion

### Stability of Diagnosis

Stability of ASD diagnosis between 3 and 7 years was moderate. Ten HR children met criteria at both time‐points (“Stable diagnosis”), 3 children who met criteria at age 3 did not at 7 (“Lost diagnosis”), and 5 children showed the opposite pattern (“Later diagnosed”). The “Lost diagnosis” subgroup had high ADOS scores, moderate ADI‐R scores and average developmental abilities at 3 years (Table S1), but by age 7 showed ADI‐R and ADOS scores similar to the HR “Never diagnosed” subgroup and a high‐average mean IQ (FSIQ = 118) (Table S2). Two children who “Lost diagnosis” were considered clinically to show traits of ASD at 7 years but did not reach the clinical threshold for ASD. The remaining “Lost diagnosis” child was considered by the research team and parents to be typically developing at 7, and whilst parents had reported considerable concerns at age 3, they had no concerns at age 7. The “Later diagnosed” subgroup had low ADI scores, ADOS scores similar to the HR “Never diagnosed” subgroup, and high developmental and adaptive ability at age 3 (Table S1). At age 7, however, their ADI and ADOS scores were high and adaptive behaviour was low, similar to the “Stable diagnosis” group, despite having high‐average intelligence (Table S2). A similar pattern of findings has been reported for later‐diagnosed high‐risk children [Brian et al., 2015] and in clinical samples [Davidovitch, Levit‐Binnun, Golan & Manning‐Courtney, 2015]. The “Later diagnosed” subgroup also had high ADHD scores and the highest anxiety scores at 7 (Table S2).

It is known from clinically referred samples that diagnostic stability from toddlerhood to mid‐childhood of more narrowly defined autism is relatively good [e.g., Charman et al., 2005; Lord et al., 2006]. However, for broader ASD stability from the preschool years to mid‐childhood is only moderate [Rondeau et al., 2011; Woolfenden, Sarkozy, Ridley, & Williams, 2012]. Stability might be expected to be lower in familial high‐risk samples compared to clinically referred samples where parents and/or professionals have explicit concerns about a child's development. Diagnostic decision‐making is a complex process in which measurement error and some degree of uncertainty is to be expected [Constantino & Charman, 2016]. In clinically referred cohorts, across the preschool to mid‐childhood age‐range levels of symptoms are relatively stable but there are some children whose symptoms lessen over time [termed “improvers” in Gotham, Pickles, & Lord, 2012] and others whose symptoms worsen [see also Fountain, Winter & Bearman, 2012]. In common with other behaviourally defined disorders [Tandon, Tillman, Agrawal & Luby, 2016]—alongside the aforementioned measurement error—some children will inevitably cross a diagnostic threshold or boundary as symptoms fluctuate over time. This is compounded in relation to ASD by the commonly co‐occurring conditions, including ADHD and anxiety, which also wax‐and‐wane in severity over development. These factors may have added to diagnostic instability across the 3 and 7 year diagnostic assessments, notwithstanding the use of standard diagnostic instruments by an experienced team. Other contributing factors include possible use of interventions for ASD (although none of the “Lost diagnosis” children had received specific interventions) and changes in diagnostic criteria used for 3‐year and 7‐year diagnoses (ICD‐10 at 3 and DSM‐5 at 7). Given the small sample sizes of these diagnostic subgroups and the limitation in the current study that the diagnostic assessments at age 3 and 7 years were not independent our ability to interpret these findings is limited and will only be possible when larger samples of HR siblings are followed into mid‐childhood.

### Recurrence Rate

It is important to note that the recurrence rate in our sample is higher than that of ∼19% reported in the overlapping BSRC pooled samples [Ozonoff et al., 2011; Messinger et al., 2015]. This was true at age 3 years [32%, Elsabbagh et al., 2012] and even more so at 7 years (36%), reflecting the fact that some children not diagnosed at age 3 years were considered to meet criteria at age 7. There is considerable cross‐site variability in recurrence rates and other studies have recently reported similarly elevated recurrence rates at age 2 years [29%, Elison et al., 2014] and 3 years [28%, Zwaigenbaum et al., 2016]. Our elevated recurrence rate might partially reflect the modest sample size (*n* = 53 at 3 years, *n* = 42 at 7 years) and whilste there was no *systematic* evidence of differences in retention from 3‐to‐7‐years based on 3 year autism severity or developmental data it does limit the generalisability of our findings to high‐risk siblings more generally.

### Non ASD Outcomes

As predicted, the HR‐ASD‐7 group had poorer outcomes than the other groups on several measures. Unimpaired IQ (FSIQ ≥ 70) in all but two children^2^ indicated this group mainly represented “high‐functioning” children with ASD. Nevertheless, these children showed significantly lower adaptive behaviour than the LR group, consistent with previously reported discrepancies between IQ (“capacity”) and the ability to cope in everyday life (“competency”) in ASD [Ashwood et al., 2015; Charman et al., 2011]. In line with the high co‐occurrence of ASD, anxiety and ADHD in population studies [Simonoff et al., 2008] and evidence for genetic overlap between these disorders [Hallett et al., 2013; Ronald et al., 2008; Tick et al., 2016], the HR‐ASD‐7 group had significantly higher ADHD and anxiety scores than LR children. Further, a large proportion of this group scored in the clinical range on the Hyperactive/Impulsive (53%) and Inattentive (33%) ADHD domains and the SCAS Total Anxiety (27%) measure. The pattern of findings in the HR‐ASD‐7 group demonstrates that these children experience a range of difficulties beyond ASD by early school‐age and highlights the need to assess the longer‐term and broader clinical outcomes of HR infants who develop ASD, which have only been reported in one previous study [Gillespie‐Lynch et al., 2015]. Our findings also have implications for understanding co‐occurring psychopathology in ASD more generally. In non‐high‐risk ASD samples, anxiety disorders (∼40%) and ADHD (∼30%) are two of the most common co‐occurring psychiatric conditions [Simonoff et al., 2008]. Our findings indicate that similar rates of ADHD and anxiety may co‐occur with ASD in non‐clinically referred samples [see Tick et al., 2016 for similar findings in a population‐based twin sample]. This is important since comorbidities may be over‐represented in clinically‐referred samples.

Contrary to our hypotheses, there was limited evidence of the BAP at the group level in the HR children without ASD. The HR‐Non‐ASD‐7 group had average IQ and did not differ significantly from the LR group in social‐communication abilities or language, although they did have higher RRB ADOS scores and lower adaptive behaviour. Warren et al. [2012] also found elevated RRB scores in their group of non‐ASD HR siblings, suggesting such children might be particularly vulnerable to difficulties in this symptom domain. Other school‐age follow‐up studies have reported social‐communication impairments in a proportion of non‐ASD HR children [Gamliel et al., 2009; Miller, Iosif, et al., 2015], as have cross‐sectional studies of school‐age non‐ASD siblings [Constantino et al., 2006]. Consistent with those studies, we did identify a sub‐set of the HR‐Non‐ASD‐7 siblings who showed subclinical ASD traits, defined as scoring above clinical cut‐offs for ASD on either the ADOS or ADI‐R but not meeting clinical criteria for ASD (HR‐Atypical‐7 group, see Supporting Information). We investigated whether this subgroup also showed problems with intellectual ability, language, and adaptive behaviour, but, in contrast to our predictions, found no evidence for this. The HR‐Atypical‐7 group did not differ from LR controls or typically developing HR non‐ASD siblings (HR‐Typical‐7 group) on FSIQ, ABC, or CELF language variables (Table S4), indicating they were functioning as well as their peers without ASD traits.

In terms of emotional and behavioural problems, as a group the HR‐Non‐ASD‐7 children did not show raised ADHD symptoms compared to the LR controls. However, 20–30% of the HR‐Non‐ASD‐7 group scored above the clinical threshold on the Conners indices, about double the rate in the LR controls, indicating there is a higher risk for inattention/hyperactivity difficulties in non‐ASD HR children than LR children. The elevated Separation Anxiety scores and higher rates of anxiety scores in the clinical range in the HR‐Non‐ASD‐7 group than in the LR controls indicates these children had difficulties with anxiety. This group also had (non‐significantly) higher mean scores on Total and Generalised Anxiety than the LR controls (Table 2), which may have reached significance in a larger sample given the large effect sizes for these comparisons (Table S3). These findings are consistent with Miller, Iosif, et al. [2015] who found that a proportion of non‐ASD HR infants showed raised anxiety/depression or ADHD problems at school‐age. Warren et al [2012] found no evidence of elevated internalising or externalising scores at school‐age in non‐ASD HR siblings, but they collapsed across symptom categories rather than examining anxiety/internalising and ADHD/externalising domains separately. In family studies around 12% of non‐ASD siblings in simplex families with a child with ASD have an ADHD diagnosis, rising to 18% in multiplex families [Oerlemans et al., 2015]. Anxiety disorders have also been reported at higher rates in older family members of individuals with an ASD diagnosis than controls [Bolton, Pickles, Murphy, & Rutter, 1998; Piven & Palmer, 1999].

We examined whether the subgroup of non‐ASD HR children with atypical outcomes showed raised ADHD and anxiety scores compared to the typically developing HR and LR children. In contrast to our predictions, we found that the HR‐Atypical‐7 group had comparable ADHD and anxiety scores to the HR‐Typical‐7 and LR groups (Table S4). This suggests that the proportion of HR non‐ASD children scoring in the clinical range on the ADHD and anxiety measures does not correspond to the proportion of HR non‐ASD siblings with elevated ASD traits. Examining early‐life predictors of later ADHD and anxiety symptoms might help identify this subgroup of children in future. Note that our definition of “atypical outcomes” in some HR children overlaps with but also differs from that adopted in previous studies. We used scores above the clinical cut‐off on the ADOS‐2 and ADI‐R to identify this group but only in children who were clinically not judged to meet diagnostic criteria for ASD. This is consistent with some previous reports [e.g., Landa, Gross, Stuart, & Faherty, 2013; Landa, Holman, & Garrett‐Mayer, 2007] but is stricter than in some studies where scores one point [e.g., Charman et al., 2016] or a few points [e.g., Ozonoff et al., 2014] below the clinical threshold have been used—although in all samples this includes children who scores extend into the clinical range. Unlike other groups [e.g., Ozonoff et al., 2014; Paul, Fuerst, Ramsay, Chawarska, & Klin, 2011] we did not attempt to apply a clinical judgement about possible or likely “BAP” presentations—in part because there are no agreed criteria for what these would be. If anything our stricter definition of “atypicality” might have made it more likely that we would find elevated ADHD and anxiety scores in these children but this was not the case.

Taken together with previous mixed reports of spared and impaired cognitive ability, language, social‐communication, and mood and behavioural problems in non‐ASD HR siblings at school‐age [Ben‐Yizhak et al., 2011; Drumm et al., 2015; Gamliel et al., 2009; Gillespie‐Lynch et al., 2015; Miller, Iosif, et al., 2015; Warren et al., 2012], our findings highlight the heterogeneity in outcomes of at‐risk children. It is clear from the mean IQ and Vineland scores of the HR‐Non‐ASD‐7 group (see Table 2) that they are functioning in the average ability range and, at least in our modest size sample, there was limited evidence at a group level that they were experiencing significant developmental problems. It will now be important to establish what intrinsic and extrinsic factors influence whether a high‐risk child without ASD will experience BAP characteristics or co‐occurring emotional and behavioural problems.

We examined whether sex of the HR sibling affected outcomes. In line with recent findings [Messinger et al., 2015] we found that boys had higher ADOS scores, lower adaptive communicative behaviour and lower levels of Generalised Anxiety than girls. In the LR group only, girls had higher FSIQ scores than boys. There were no sex‐by‐group interactions on measures of ASD severity or behavioural outcomes indicating no strong sex specific liability to elevated levels of ASD, ADHD, and anxiety symptoms in HR siblings [see also Zwaigenbaum et al., 2012], although our modest sample sized means we were under‐powered to detect such differences.

### Limitations

Our sample sizes for the high‐risk subgroups were modest and this should be considered when interpreting our findings. The recurrence rate in our sample is high compared to the 3‐year estimates from the large pooled BSRC dataset which may limit the generalisability of findings to high‐risk siblings more broadly. Further, although there are many strengths to our design, including the use of well‐established and validated instruments to measure ASD and other phenotypic characteristics, there are also limitations in the implementation of the design, including a lack of independence between the 3 year and 7 year diagnostic judgements. In common with other high‐risk sibling studies our HR children—including those with ASD—have high IQs compared to clinical samples of children with ASD, which may also limit generalisability. However, the clinical relevance of our findings is supported by the large effect sizes of significant group differences. Finally, although we carefully assessed ASD symptomatology with observer‐ and parent‐rated measures we were unable to conduct similarly in‐depth assessments of ADHD and anxiety and relied upon parent‐report questionnaires. These questionnaires are well‐validated and relate to DSM criteria for ADHD and anxiety disorders, but future work should undertake multi‐informant assessments to obtain a more reliable characterisation of symptoms and co‐occurring diagnoses.

## Supporting information


**Table S1** ASD, developmental level and adaptive behaviour scores at 3 years in high‐risk grouped by diagnostic change from 3‐7 years
**Table S2** ASD, developmental level and adaptive behaviour scores at 7 years in high‐risk grouped by diagnostic change from 3‐7 years
**Table S3** Cohen's *d* effect sizes for significant and non‐significant pairwise group contrasts
**Table S4** Group characteristics and ASD symptomatology at 7 years. Means (SD) are presented by ASD outcome group.

## Footnotes

1 5 DAWBA and 5 SCQ missing.

2 One HR‐ASD‐7 child could not complete the WASI‐II due to intellectual impairment; a second HR‐ASD‐7 child had FSIQ < 70.
