# Supplementary material for: Mid‐childhood outcomes of infant siblings at familial high‐risk of autism spectrum disorder
Source: Autism Res. 2016 Nov 29;10(3):546–57. doi: 10.1002/aur.1733 (PMC5408391; doi:10.1002/aur.1733)
Supplement: Supplementary file 1 — Table S1 ASD, developmental level and adaptive behaviour scores at 3 years in high‐risk grouped by diagnostic change from 3‐7 years Table S2 ASD, developmental level and adaptive behaviour scores at 7 years in high‐risk grouped by diagnostic change from 3‐7 years Table S3 Cohen's d effect sizes for significant and non‐significant pairwise group contrasts Table S4 Group characteristics and ASD symptomatology at 7 years. Means (SD) are presented by ASD outcome group. [file AUR-10-546-s001.docx]

Supplementary Materials

*Differences between the HR groups who changed diagnostic category between 3 years and 7 years*

*3 year scores by diagnostic change*

Scores at 3 years are presented by diagnostic change group in Table S1. The MANOVA on ADI variables for the four diagnostic change groups revealed significant group effects for Social (*F*(3, 38) = 14.94, *p* < .001, *η^2^* = .541), Communication (*F*(3, 38) = 6.80, *p* = .001, *η^2^* = .349), and RRB (*F*(3, 38) = 9.15, *p* < .001, *η^2^* = .420). Social scores were significantly higher in Stable Diagnosed than Never Diagnosed (*p* < .001, *d* = 2.23) and Later Diagnosed (*p* < .001, *d* = 3.14) (all other *p* > .09). Communication scores were significantly higher in Stable Diagnosed than Never Diagnosed (*p* = .001, *d* = 1.44) and Later Diagnosed (*p* = .008, *d* = 2.07) (all other *p* > .15). RRB scores were significantly higher in Stable Diagnosed than Never Diagnosed (*p* < .001, *d* = 1.58) and Later Diagnosed (*p* = .01, *d* = 1.61) (all other *p* > .20). The MANOVA on ADI variables comparing two groups (Stable and Later diagnosed) revealed significantly higher scores in Stable Diagnosed than Later Diagnosed for Social (*F*(1, 13) = 26.36, *p* < .001, *η^2^* = .670), Communication (*F*(1, 13) = 10.93, *p* = .006, *η^2^* = .457), and RRB (*F*(1, 13) = 6.64, *p* = .02, *η^2^* = .338).

The MANOVA on ADOS variables for the four diagnostic change groups revealed a significant group effect for Total CSS (*F*(3, 38) = 3.46, *p* = .03, *η^2^* = .215) and a trend for RRB (*F*(3, 38) = 2.69, *p* = .06, *η^2^* = .175), but no difference for Social Affect (*F*(3, 38) = 1.73, *p* = .18, *η^2^* = .120). Total CSS scores were significantly higher in Stable Diagnosed than Never Diagnosed (*p* = .05, *d* = .96) (all other *p* > .16). RRB scores did not differ between groups in pairwise contrasts (all *p* > .10). The MANOVA on ADOS variables comparing Stable and Later diagnosed revealed no group differences (all *p* > .12).

The four diagnostic change groups differed significantly in SRS-2 (*F*(3, 37) = 5.18, *p* = .004, *η^2^* = .296) scores, which reflected significantly higher scores in Stable Diagnosed than Never Diagnosed (*p* = .003, *d* = 1.27); there was a trend for higher SRS-2 scores in Stable Diagnosed than Lost Diagnosis (*p* = .07, *d* = 1.74) (all other *p* > .20). Univariate ANOVAs comparing the Stable and Later Diagnosed groups revealed a trend for higher SCQ scores in Stable than Later Diagnosed (*F*(1, 12) = 3.99, *p* = .07, *η^2^* = .249), but no difference in SRS-2 scores (*F*(1, 12) = 2.82, *p* = .11, *η^2^* = .190).

There was a marginal effect of group on ELC (*F*(3, 38) = 2.82, *p* = .05, *η^2^* = .182), which reflected a trend for lower ELC in Stable than Never Diagnosed (*p* = .06, *d* = .87). The ANOVA comparing Stable and Later Diagnosed groups revealed a trend for lower ELC in Stable Diagnosed (*F*(1, 13) = 3.63, *p* = .08, *η^2^* = .218). Adaptive behaviour did not differ significantly between the four groups (*F*(3, 38) = 1.94, *p* = .14, *η^2^* = .133), or between the Stable and Later Diagnosed groups (*F*(1, 13) = 2.63, *p* = .13, *η^2^* = .168).

**Table S1**

ASD, developmental level and adaptive behaviour scores at 3 years in high-risk grouped by diagnostic change from 3-7 years

|  | **High Risk Grouped by Diagnostic Change** | | | |
| --- | --- | --- | --- | --- |
| **3 year scores** | **Never Diagnosed**  **N = 24^*^** | **Later Diagnosed**  **N = 5^*^** | **Lost Diagnosis**  **N = 3** | **Stable Diagnosed**  **N = 10^*^** |
| *ADI – Social* | 2.75 (3.71) | 1.40 (1.95) | 5.67 (4.04) | 11.50 (4.12) |
| *ADI – Communication* | 2.96 (4.13) | 1.80 (1.64) | 3.67 (3.79) | 9.60 (5.06) |
| *ADI – RRB* | 0.71 (1.20) | 0.80 (0.84) | 2.67 (2.08) | 3.70 (2.41) |
| *ADOS – CSS Total* | 3.42 (2.54) | 3.60 (2.41) | 6.67 (0.58) | 5.90 (2.60) |
| *ADOS – SA CSS* | 4.42 (2.77) | 4.80 (2.95) | 7.33 (0.58) | 6.10 (2.51) |
| *ADOS – RRB CSS* | 4.04 (2.56) | 4.80 (2.28) | 7.00 (1.00) | 6.20 (2.39) |
| *SCQ* | 3.75 (6.56) | 6.20 (5.07) | 5.33 (3.22) | 14.11 (7.93) |
| *SRS-2* | 45.63 (10.60) | 49.80 (8.59) | 43.00 (3.61) | 62.67 (15.68) |
| *Mullen ELC* | 109.83 (16.91) | 116.20 (18.21) | 99.00 (25.87) | 89.80 (27.87) |
| *Vineland ABC* | 98.96 (10.24) | 104.40 (7.40) | 92.33 (4.16) | 90.40 (18.28) |

* Ns are lower for some measures due to missing data. *ADI* = Autism Diagnostic Interview – Revised; *ADOS CSS Total, SA, RRB* = Autism Diagnostic Observation Schedule Calibrated Severity Scores for Total, Social Affect and restricted and repetitive behaviours; *SRS-2* = Social Responsiveness Scale – 2 (Preschool version); *RBS-R* = Repetitive Behavior Scale – Revised; *SSP* = Short Sensory Profile. *Mullen ELC* = Mullen Scales of Early Learning Early Learning Composite. *Vineland ABC* = Vineland Adaptive Behavior Scales – II Adaptive Behavior Composite.

*7 year scores by diagnostic change*

Scores at 7 years are presented by diagnostic change group in table S2. The MANOVA on ADI variables for the four diagnostic change groups revealed significant group effects for Social (*F*(3, 37) = 9.71, *p* < .001, *η^2^* = .440), Communication (*F*(3, 37) = 5.62, *p* = .003, *η^2^* = .313), and RRB (*F*(3, 37) = 11.64, *p* < .001, *η^2^* = .485). Social scores were significantly lower in Never Diagnosed than Stable Diagnosed (*p* < .001, *d* = 1.93) and Later Diagnosed (*p* = .02, *d* = 1.50), and there was a trend for lower scores in Lost Diagnosis than Stable Diagnosed (*p* = .07, *d* = 2.30). Communication scores were significantly lower in Never Diagnosed than Stable Diagnosed (*p* = .009, *d* = 1.31) and Later Diagnosed (*p* = .04, *d* = 1.29); the other groups did not differ (all *p* > .18). RRB scores were significantly lower in Never Diagnosed than Stable Diagnosed (*p* < .001, *d* = 1.78) and Later Diagnosed (*p* = .007, *d* = 2.26); RRB scores were also significantly lower in Lost Diagnosis than Stable Diagnosed (*p* = .05, *d* = 1.68). The MANOVA on ADI variables comparing the two groups (Stable and Later diagnosed) revealed no group differences (all *p* > .38).

MANOVA on ADOS variables for the four diagnostic change groups revealed significant group effects for Total CSS (*F*(3, 38) = 6.54, *p* = .001, *η^2^* = .340), Social Affect (*F*(3, 38) = 9.89, *p* < .001, *η^2^* = .438), and RRB (*F*(3, 38) = 3.63, *p* = .02, *η^2^* = .223). Total CSS scores were significantly lower in Never Diagnosed than Stable (*p* = .005, *d* = 1.15) and Later (*p* = .02, *d* = 1.56) Diagnosed (all other *p* > .15). Social Affect scores were significantly lower in Never Diagnosed than Stable (*p* < .001, *d* = 1.54) and Later (*p* = .002, *d* = 1.90) Diagnosed (all other *p* > .14). RRB scores were significantly lower in Never Diagnosed than Stable (*p* = .02, *d* = 1.17) Diagnosed (all other *p* > .23). The MANOVA on ADOS variables comparing the two groups (Stable and Later diagnosed) revealed no group differences (all *p* > .69).

The four diagnostic change groups differed significantly in SCQ (*F*(3, 35) = 7.03, *p* = .001, *η^2^* = .376) and SRS-2 (*F*(3, 35) = 5.87, *p* = .003, *η^2^* = .362) scores. SCQ scores were significantly lower in Never Diagnosed than Stable (*p* = .004, *d* = 1.45) and Later (*p* = .009, *d* = 1.26) Diagnosed (all other *p* > .15). SRS-2 scores were significantly lower in Never Diagnosed than Stable Diagnosed (*p* = .05, *d* = 1.01) and Later Diagnosed (*p* = .01, *d* = 1.58), but higher than in Lost Diagnosis (*p* = .04, *d* = .81) (all other *p* > .11). The Stable and Later Diagnosed groups did not differ in SCQ (*F*(1, 12) = 0.78, *p* = .79, *η^2^* = .006) or SRS-2 (*F*(1, 11) = 0.40, *p* = .54, *η^2^* = .035) scores.

IQ did not differ between the four diagnostic change groups (*F*(3, 37) = 0.38, *p* = .77, *η^2^* = .030), or between the Stable and Later Diagnosed groups (*F*(1, 12) = 0.05, *p* = .82, *η^2^* = .004). Adaptive behaviour did differ significantly between the four groups (*F*(3, 37) = 4.13, *p* = .01, *η^2^* = .251), which reflected significantly higher ABC scores in the Never Diagnosed group than the Stable Diagnosed (*p* = .04, *d* = .75), and lower scores in the Never Diagnosed group than the Lost Diagnosis (*p* = .04, *d* = 1.47) group (all other *p* > .16). The Stable and Later Diagnosed groups did not differ in ABC scores (*F*(1, 13) = 0.10, *p* = .75, *η^2^* = .008).

ADHD and anxiety: the MANOVA on ADHD scores revealed a trend for an effect of group for Hyperactive/Impulsive scores (*F*(3, 36) = 2.70, *p* = .06, *η^2^* = .184), which reflected a tendency for higher scores in the Stable diagnosis group than in the Lost diagnosis group (*p* = .087). Inattentive scores did not differ between groups (*p* = .20). Total anxiety scores did not differ significantly between groups (*p* = .26).

**Table S2**

ASD, developmental level and adaptive behaviour scores at 7 years in high-risk grouped by diagnostic change from 3-7 years

|  | **High Risk Grouped by Diagnostic Change** | | | |
| --- | --- | --- | --- | --- |
| **7 year scores** | **Never Diagnosed**  **N = 24^*^** | **Later Diagnosed**  **N = 5^*^** | **Lost Diagnosis**  **N = 3** | **Stable Diagnosis**  **N = 10*** |
| *ADI – Social* | 4.04 (5.48) | 11.60 (4.56) | 5.33 (2.31) | 14.00 (4.80) |
| *ADI – Communication* | 4.25 (4.67) | 10.60 (5.13) | 4.00 (1.73) | 10.33 (4.58) |
| *ADI – RRB* | 0.58 (1.41) | 3.20 (.84) | 1.00 (1.00) | 3.78 (2.11) |
| *ADOS – Total CSS* | 2.42 (1.50) | 5.80 (2.68) | 2.33 (1.16) | 5.40 (3.34) |
| *ADOS – SA CSS* | 2.96 (1.60) | 7.00 (2.55) | 3.67 (2.08) | 6.40 (2.72) |
| *ADOS – RRB CSS* | 3.04 (2.84) | 5.80 (2.95) | 3.33 (4.04) | 6.30 (2.71) |
| *SRS-2 ^T-score^* | 52.37 (11.74) | 80.00 (21.77) | 45.33 (3.79) | 71.63 (24.23) |
| *WASI FSIQ* | 107.96 (12.76) | 111.6 (7.60) | 118.3 (18.01) | 108.78 (26.63) |
| *Vineland ABC* | 102.22 (12.67) | 88.40 (14.55) | 116.33 (4.93) | 91.20 (16.57) |
| *Conners Hyp/Imp* | 57.05 (16.10) | 67.40 (11.46) | 42.67 (3.06) | 67.90 (17.87) |
| *Conners Inattentive* | 56.50 (12.90) | 62.20 (8.04) | 44.33 (2.52) | 62.50 (17.80) |
| *SCAS Total Anxiety* | 17.91 (8.56) | 31.60 (26.75) | 23.33 (5.13) | 23.50 (18.28) |

* Ns are lower for some measures due to missing data. *ADI* = Autism Diagnostic Interview – Revised; *ADOS CSS Total, SA, RRB* = Autism Diagnostic Observation Schedule Calibrated Severity Scores for Total, Social Affect and restricted and repetitive behaviours; *SRS-2* = Social Responsiveness Scale – 2; *RBS-R* = Repetitive Behavior Scale – Revised; *SSP* = Short Sensory Profile. *WASI FSIQ* = Wechsler Abbreviated Scale of Intelligence – II Full-Scale IQ. *Vineland ABC* = Vineland Adaptive Behavior Scales – II Adaptive Behavior Composite. *Conners Hyp/Imp and Inattentive* = Conners 3 T-scores for Hyperactive/Impulsive and Inattentive symptoms. *SCAS* = Spence Children’s Anxiety Scale.

**Table S3** Cohen’s *d* effect sizes for significant and non-significant pairwise group contrasts

| **7-year outcome measure** | **LR vs.**  **HR-Non-ASD-7** | **LR vs.**  **HR-ASD-7** | **HR-Non-ASD-7 vs. HR-ASD-7** |
| --- | --- | --- | --- |
| *ADI Social* | ----- | ----- | 1.78* |
| *ADI Communication* | ----- | ----- | 1.33* |
| *ADI RRB* | ----- | ----- | 1.89* |
| *ADOS Total CSS* | 0.58 | 2.08* | 1.69 |
| *ADOS SA CSS* | 0.47 | 2.02* | 1.69 |
| *ADOS RRB CSS* | 0.93* | 2.54* | 1.12 |
| *SRS-2* | 0.74 | 1.77* | 1.24* |
| *RBS-R* | 0.44 | 1.21* | 1.00* |
| *SSP* | 0.37 | 1.21* | 0.85* |
| *WASI-II FSIQ* | 0.75* | 0.42 | 0.10 |
| *WASI-II VCI* | 0.62 | 0.46 | 0.03 |
| *WASI-II PRI* | 0.69 | 0.05 | 0.47 |
| *Vineland ABC* | 0.81* | 1.69* | 0.85* |
| *Vineland Socialization* | 0.63 | 2.04* | 1.39* |
| *Vineland Daily Living* | 0.77* | 1.54* | 0.93* |
| *Vineland Communication* | 0.37 | 0.81* | 0.45 |
| *CELF CFD* | 0.32 | 0.66 | 0.32 |
| *CELF RS* | 0.57 | 0.08 | 0.51 |
| *Conners Hyp/Imp* | 0.35 | 1.13* | 0.67 |
| *Conners Inattentive* | 0.47 | 0.90* | 0.42 |
| *SCAS Separation Anxiety* | 0.77* | 1.00* | 0.39 |
| *SCAS OCD* | 0.26 | 0.64* | 0.48 |
| *SCAS Social* | 0.53 | 0.54 | 0.22 |
| *SCAS Physical Injury Fears* | 0.22 | 0.63 | 0.43 |
| *SCAS Panic/Agoraphobia* | 0.22 | 0.72* | 0.66 |
| *SCAS Generalised Anxiety* | 0.72 | 0.96* | 0.47 |
| *SCAS Total Anxiety* | 0.72 | 0.89* | 0.52 |

* significant group difference (*p* < 0.05 with Tukey’s HSD correction applied). *ADI* = Autism Diagnostic Interview – Revised. *ADOS Total, SA, RRB CSS* = Autism Diagnostic Observation Schedule Calibrated Severity Scores for Total, Social Affect and restricted and repetitive behaviours. *SRS-2* = Social Responsiveness Scale – 2; *RBS-R* = Repetitive Behavior Scale – Revised; *SSP* = Short Sensory Profile. *WASI-II FSIQ, VCI, PRI* = Wechsler Abbreviated Scale of Intelligence – II Full-Scale IQ, Verbal Comprehension Index, Perceptual Reasoning Index. *Vineland ABC, Socialization, Daily Living, Communication* = Vineland Adaptive Behavior Scales – II Adaptive Behavior Composite (ABC) and Standard Scores for Socialization, Daily Living and Communication domains. *CELF CFD RS* = Clinical Evaluation of Language Fundamentals – 4^th^ Edition Scaled Scores for Concepts and Following Directions (CFD) and Recalling Sentences (RS). *Conners Hyp/Imp and Inattentive* = Conners 3 T-scores for Hyperactive/Impulsive and Inattentive symptoms. *SCAS* = Spence Children’s Anxiety Scale.

*Differences in school-age outcomes between HR children with ASD, Atypical and Typical development and LR controls*

Following the approach taken in previous school-age follow-up studies (Gamliel et al., 2009; Miller et al., 2015b), we explored differences in outcome variables between HR children with ASD (HR-ASD-7 group), HR children with atypical development (HR-Atypical-7 group), HR children with typical development (HR-Typical-7 group) and LR controls. The HR-ASD-7 group (n = 15) and the LR group (n = 37) were as reported in the main text. The HR-Atypical-7 group included 7 HR children who did not meet DSM-5 criteria for ASD at age 7 but scored above clinical cut-offs for ASD on either (but not both) the ADOS (n = 5) or ADI-R (n = 2). Two of the three children who ‘lost diagnosis’ from age 3 were included in the HR-Atypical-7 group as, although we excluded them from the main analysis because we did not feel it was appropriate to include them in the HR-Non-ASD-7 group, they met criteria for atypical development at age 7. The HR-Typical-7 group included 19 HR children with below-threshold scores on the ASD instruments and who had IQ scores > 70. In line with the BAP concept, we expected to find poorer outcomes (lower IQ, adaptive behaviour and language; higher ADHD and anxiety) in the HR-Atypical-7 group than the HR-Typical-7 and LR groups, who would not differ from one another.

Group means for the key outcome variables and statistical test results are displayed in Table S4. MANOVA revealed significant group effects for ADI-R Social, Communication, and RRB scores. The HR-ASD-7 and HR-Atypical-7 groups had significantly higher Social and Communication scores than the HR-Typical-7 group (all *p* < .01, all *d* > 1.11). ADI-R RRB scores were significantly higher in the HR-ASD-7 group than the HR-Atypical-7 and HR-Typical-7 groups (all *p* < .003, all *d* > 1.23), who did not differ from one another. MANOVA revealed significant group effects for ADOS-2 Social Affect, RRB, and Total CSS. The HR-ASD-7 group had higher scores on all three variables than the LR and HR-Typical-7 groups and higher Total CSS than the HR-Atypical-7 group (all *p* < .03, all *d* > .093). The HR-Atypical-7 group had higher scores on all three variables than LR controls and higher RRB and Total CSS than the HR-Typical-7 group (all *p* < .02; all *d* > 1.16). WASI-II FSIQ and CELF language variables did not differ between groups. The groups differed significantly on the Vineland ABC, reflecting lower scores in the HR-ASD-7 group than the HR-Typical-7 and LR groups (all *p* < .004; all *d* > 1.00). The remaining group pairs did not differ. The HR-ASD-7 group had higher scores on both ADHD domains than the LR controls (both *p* < .01; both *d* > 0.90), while the remaining group pairs did not differ. SCAS Total Anxiety scores differed significantly between groups, reflecting higher scores in the HR-ASD-7 group than the LR controls (*p* = .001, *d* = .89). The remaining group pairs did not differ.

**Table S4** Group characteristics and ASD symptomatology at 7 years. Means (SD) are presented by ASD outcome group.

| **7-year Outcome Measure** | **LR**  **N = 37^*^** | **HR-Non-ASD-7**  **N = 19^*^** | **HR-Atypical-7**  **N = 7** | **HR-ASD-7**  **N = 15^*^** | **MANOVA/ANOVA Group effects** |
| --- | --- | --- | --- | --- | --- |
| *Age (months)* | 89.34 (4.81) | 91.53 (6.10) | 90.43 (6.40) | 89.13 (6.53) | n/s |
| *Sex (male: female)* | 15:22 | 5:14 | 2:5 | 7:8 | n/s |
| *ADI Social* | Not completed | 2.11 (2.58)^a^ | 9.86 (6.82)^b^ | 13.14 (4.69)^b^ | *F*(2, 37) = 28.02, *p* < .001, *η^2^* = .602 |
| *ADI Communication* | Not completed | 2.79 (2.20)^a^ | 8.29 (6.63)^b^ | 10.43 (4.59)^b^ | *F*(2, 37) = 14.82, *p* < .001, *η^2^* = .445 |
| *ADI RRB* | Not completed | 0.37 (0.90)^a^ | 1.14 (2.19)^a^ | 3.57 (1.74)^b^ | *F*(2, 37) = 18.94, *p* < .001, *η^2^* = .506 |
| *ADOS – CSS Total* | 1.70 (1.19)^a^ | 2.00 (0.82)^a^ | 4.14 (1.57)^b^ | 6.33 (2.92)^c^ | *F*(3, 70) = 30.59, *p* < .001, *η^2^* = .567 |
| *ADOS CSS SA* | 2.18 (1.70)^a^ | 2.53 (1.17)^ab^ | 4.57 (1.90)^bc^ | 6.60 (2.59)^c^ | *F*(3, 70) = 22.70, *p* < .001, *η^2^* = .493 |
| *ADOS CSS RRB* | 1.12 (0.70)^a^ | 2.26 (2.23)^a^ | 5.57 (3.36)^b^ | 6.13 (2.70)^b^ | *F*(3, 70) = 27.08, *p* < .001, *η^2^* = .537 |
| *WASI-II FSIQ* | 117.06 (11.61) | 108.89 (13.03) | 105.43 (10.28) | 109.79 (21.36) | n/s |
| *Vineland ABC* | 110.53 (6.98)^a^ | 103.89 (11.62)^a^ | 101.14 (15.45) | 90.27 (15.46)^b^ | *F*(3, 70) = 11.66, *p* < .001, *η^2^* = .333 |
| *CELF CFD* | 12.71 (2.20) | 12.33 (2.53) | 11.14 (3.76) | 10.83 (3.38) | n/s |
| *CELF RS* | 12.79 (3.01) | 11.53 (2.20) | 11.14 (1.87) | 12.55 (2.77) | n/s |
| *Conners Hyp/Imp* | 52.16 (11.58)^a^ | 56.12 (15.93) | 54.71 (17.56) | 67.73 (15.58)^b^ | *F*(3, 72) = 4.44, *p* = .006, *η^2^* = .156 |
| *Conners Inattentive* | 51.22 (9.40)^a^ | 55.65 (10.43) | 55.43 (18.16) | 62.40 (14.91)^b^ | *F*(3, 72) = 3.25, *p* = .03, *η^2^* = .119 |
| *SCAS Total Score* | 12.22 (7.27)^a^ | 18.22 (9.66) | 17.86 (2.55) | 26.20 (20.86)^b^ | *F*(3, 72) = 5.33, *p* = .002, *η^2^* = .182 |

Groups marked with different superscript letters (a, b, c) differed significantly with Tukey’s HSD correction applied (*p* < .05). * Group sizes are smaller for some variables due to missing data. *ADI* = Autism Diagnostic Interview – Revised*. ADOS* = Autism Diagnostic Observation Schedule; CSS = Calibrated Severity Score; SA = Social Affect. *WASI-II FSIQ* = Wechsler Abbreviated Scale of Intelligence – II Full-Scale IQ. *Vineland ABC* = Vineland Adaptive Behavior Scales – II Adaptive Behavior Composite. *CELF CFD RS* = Clinical Evaluation of Language Fundamentals – 4^th^ Edition Scaled Scores for Concepts and Following Directions (CFD) and Recalling Sentences (RS). *Conners Hyp/Imp and Inattentive* = Conners 3 T-scores for Hyperactive/Impulsive and Inattentive symptoms. *SCAS* = Spence Children’s Anxiety Scale (parent-rated scores for Total Anxiety).
